# Supplementary material for: Determinants of Diet Quality in Adolescents: Results from the Prospective Population-Based EVA-Tyrol and EVA4YOU Cohorts
Source: Nutrients. 2023 Dec 18;15(24):5140. doi: 10.3390/nu15245140 (PMC10746085; doi:10.3390/nu15245140)
Supplement: Supplementary file 1 [file nutrients-15-05140-s001.zip › Determinants of diet quality in adolescents_Supplement.pdf]

Determinants of diet quality in adolescents: results from the prospective population-based EVA-Tyrol and EVA4YOU cohorts

Katharina Mueller (1,2), Alex Messner (2), Johannes Nairz (3), Bernhard Winder (4), Anna Staudt (2), Katharina Stock (2), Nina Gande (2), Christoph Hochmayr (2), Benoit Bernar (5), Raimund Pechlaner (6), Andrea Griesmacher (7), Alexander E Egger (8), Ralf Geiger (3), Ursula Kiechl-Kohlendorfer (2), Michael Knoflach*(1,6), Sophia Zollner-Kiechl*(1,9) for the EVA-Tyrol and EVA4YOU Study groups.

Supplements

Assessment of dietary scores

Table S1: Alternate Healthy Eating Index (AHEI-2010) components and criteria for scoring [1]

Component	Criteria for minimum score 0	Criteria for maximum score of 10
Whole fruit	0	≥ 4 servings/d
Vegetable (excluding potatoes)	0	≥ 5 servings/d
Whole grains	0	Female: ≥ 75 g/d
		Male: ≥ 90 g/d
Red and processed meat	≥ 1.5 servings/day	0
Nuts and legumes	0	≥ 1 serving/d
Long-chain (ω-3) fats (EPA+DHA)	0	≥ 250 mg/d
Polyunsaturated fatty acids	≤ 2 % of energy	≥ 10 % of energy
Trans fats	≥ 4 % of energy	≤ 0.5 % of energy
SSBs and fruit juice	≥ 1 serving/d	0
Sodium	*Highest decile*	*Lowest decile*
	Female: ≥ 3337 mg/d	Female: ≤ 1112 mg/d
	Male: ≥ 5271 mg/d	Male: ≤ 1612 mg/d
Total	0	100

EPA, eicosapentaenoic acids; DHA, docosahexaenoic acids; SSB, Sugar-sweetened beverage.

Table S2: Dietary Approaches to Stop Hypertension (DASH)-score components and criteria for scoring [2]

Component	Criteria for minimum score 1<br>Servings/d	Criteria for maximum score of 5<br>Servings/d
Whole fruit and fruit juices	0.7	4.1
Vegetable (excluding potatoes) and vegetable juices	1.1	4.6
Whole grains	0.1	2.4
Nuts and legumes	0.3	1.5
Low-fat dairy	0.1	2.3
Sodium	2676 mg	1041 mg
Red and processed meat	1.8	0.4
Sweetened beverages	1.2	0
Total	8	40

Dietary scores and scoring in various categories

Figure S1: AHEI-2010 components and percentage of participants scoring in various categories

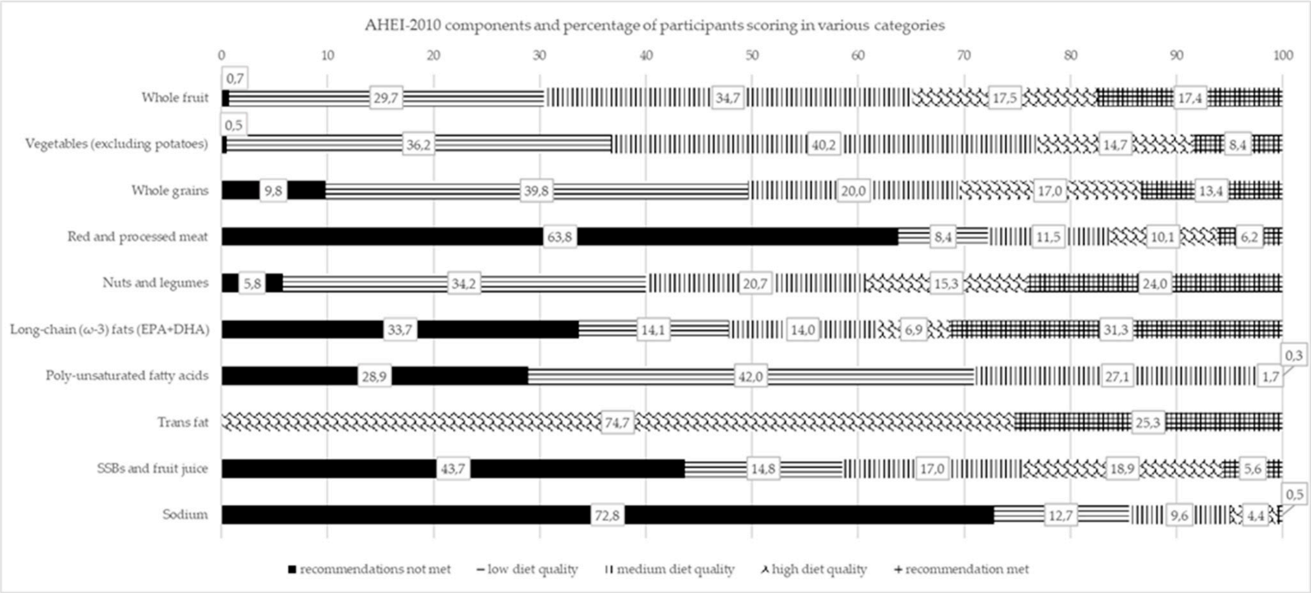

Recommendations not met, 0 points; low diet quality, 1-3 points; medium diet quality, 4-6 points; high diet quality, 7-9 points, recommendations met, 10 points.

Figure S2: DASH-score components and percentage of participants scoring in various categories

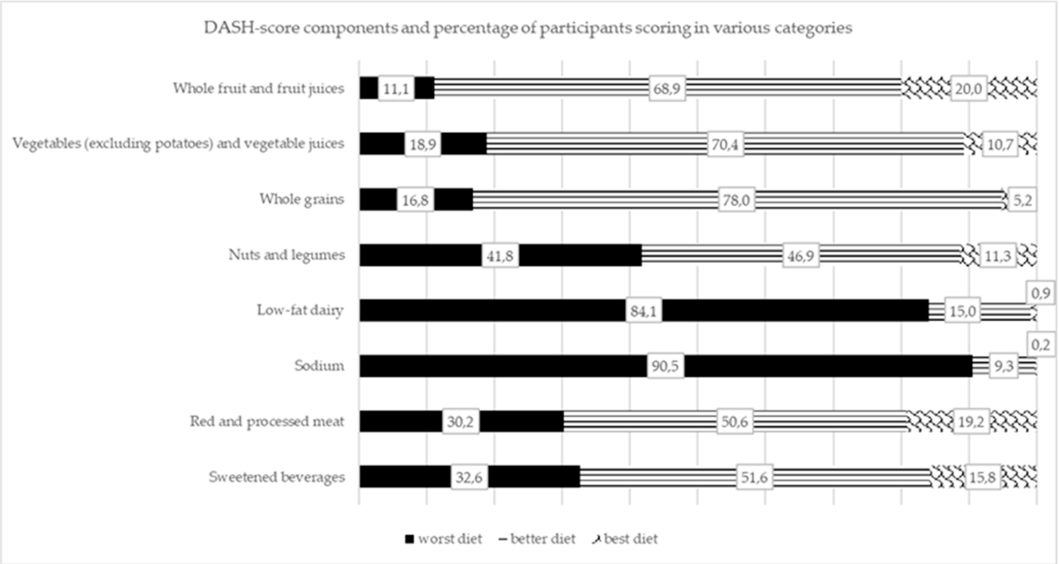

Worst diet, 1 point; better diet, 2-4 points, best diet, 5 points.

References

1. Chiuve, S.E.; Fung, T.T.; Rimm, E.B.; Hu, F.B.; McCullough, M.L.; Wang, M.; Stampfer, M.J.; Willett, W.C. Alternative dietary indices both strongly predict risk of chronic disease. *J. Nutr.* **2012**, *142*, 1009–18. doi: 10.3945/jn.111.157222.
2. Fung T.T.; Chiuve, S.E.; McCullough, M.L.; Rexrode, K.M.; Logroscino, G.; Hu, F.B. Adherence to a DASH-style diet and risk of coronary heart disease and stroke in women. *Arch. Intern. Med.* **2008**, *168*, 713–20. doi: 10.1001/archinte.168.7.713.
