# Supplementary figures and images for: Determinants of Diet Quality in Adolescents: Results from the Prospective Population-Based EVA-Tyrol and EVA4YOU Cohorts
Source: Nutrients. 2023 Dec 18;15(24):5140. doi: 10.3390/nu15245140 (PMC10746085; doi:10.3390/nu15245140)

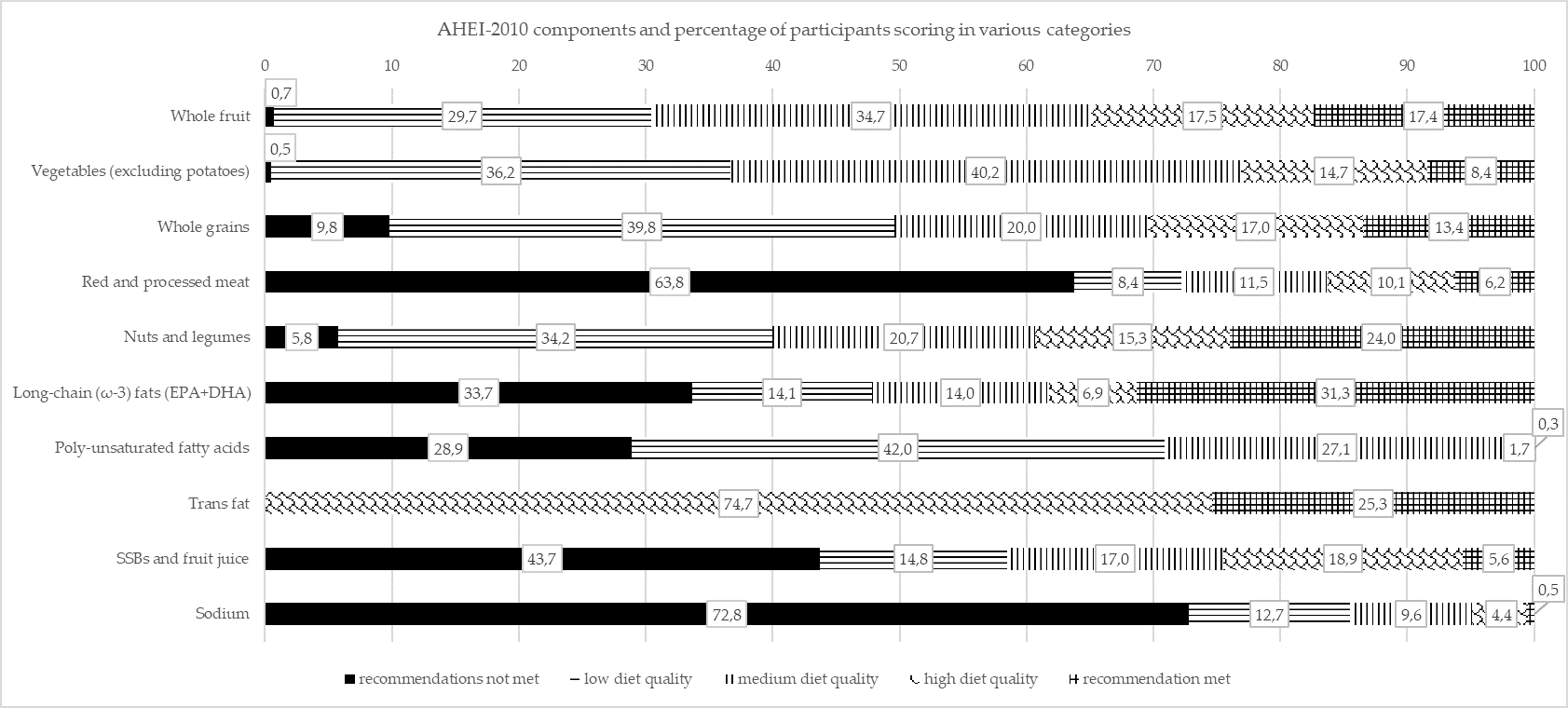

Supplement: Supplementary file 1 [file nutrients-15-05140-s001.zip › FigS1_AHEI-2010 components and percentage of participants scoring in various categories.png]

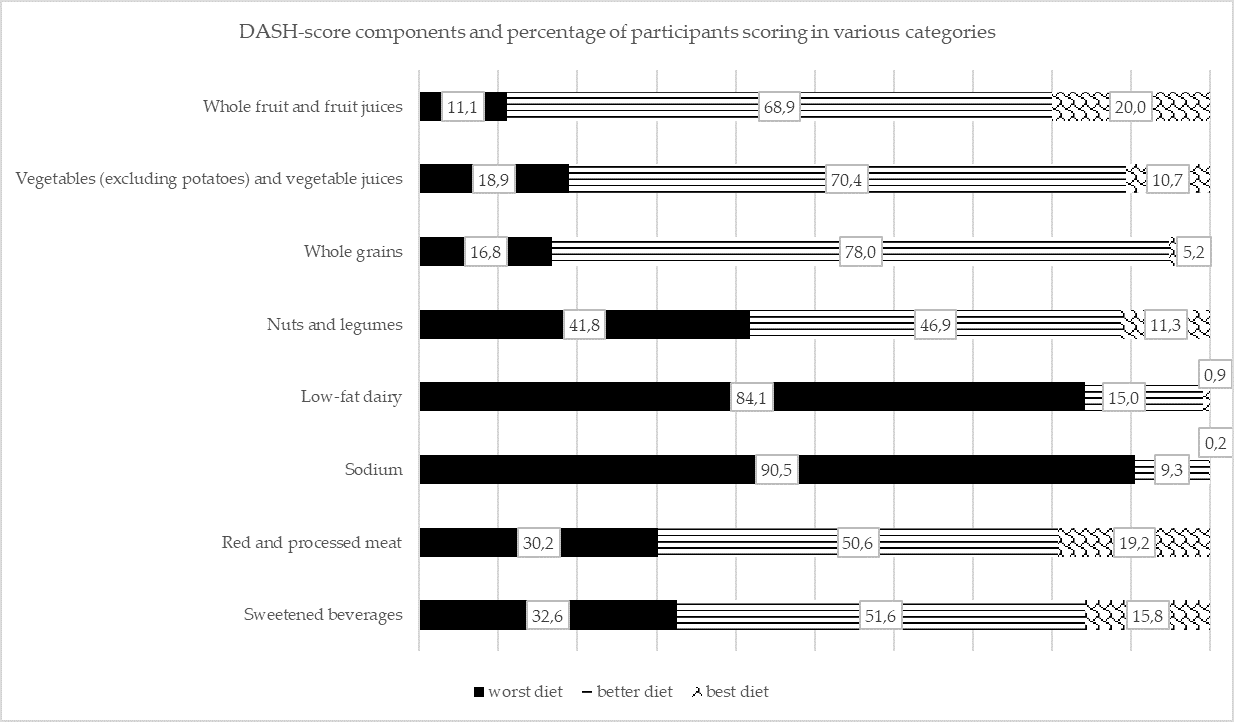

Supplement: Supplementary file 1 [file nutrients-15-05140-s001.zip › FigS2_DASHscore components and percentage of participants scoring in various categories.png]
